# Revascularization and Left Ventricular Dysfunction for ICD Eligibility

**DOI:** 10.3390/life13091940

**Published:** 2023-09-21

**Authors:** Letizia Rosa Romano, Carmen Anna Maria Spaccarotella, Ciro Indolfi, Antonio Curcio

**Affiliations:** 1 Division of Cardiology, Department of Medical and Surgical Sciences, Magna Graecia University, 88100 Catanzaro, Italy; 2 Division of Cardiology, Department of Advanced Biomedical Sciences, Federico II University, 80134 Naples, Italy

**Keywords:** revascularization, heart failure, cardiac dysfunction, implantable cardioverter–defibrillator, ischemic cardiomyopathy

## Abstract

Common triggers for sudden cardiac death (SCD) are transient ischemia, hemodynamic fluctuations, neurocardiovascular influences, and environmental factors. SCD occurs rapidly when sinus rhythm degenerates into ventricular tachycardia (VT) and/or ventricular fibrillation (VF), followed by asystole. Such progressive worsening of the cardiac rhythm is in most cases observed in the setting of ischemic heart disease and often associated with advanced left ventricular (LV) impairment. Revascularization prevents negative outcomes including SCD and heart failure (HF) due to LV dysfunction (LVD). The implantable cardioverter–defibrillator (ICD) on top of medical therapy is superior to antiarrhythmic drugs for patients with LVD and VT/VF. The beneficial effects of ICD have been demonstrated in primary prevention of SCD as well. However, yet debated is the temporal management for patients with LVD who are eligible to ICD prior to revascularization, either through percutaneous or surgical approach. Restoration of coronary blood flow has a dramatic impact on adverse LV remodeling, while it requires aggressive long-term antiplatelet therapy, which might increase complication for eventual ICD procedure when percutaneous strategy is pursued; on the other hand, when LV and/or multiorgan dysfunction is present and coronary artery bypass grafting is chosen, the overall risk is augmented, mostly in HF patients. The aims of this review are to describe the pathophysiologic benefits of revascularization, the studies addressing percutaneous, surgical or no revascularization and ICD implantation, as well as emerging defibrillation strategies for patients deemed at transient risk of SCD and/or at higher risk for transvenous ICD implantation.

## 1. Introduction

Left ventricular dysfunction (LVD) is associated with considerable morbidity and mortality and currently represents the most common cause of heart failure (HF) in the developed world. While revascularization through either percutaneous coronary interventions (PCI) or coronary artery bypass graft (CABG) has reached notable advances in the treatment of patients with acute (ACS) and chronic coronary syndromes (CCS) by drastically reducing the acute mortality, an inevitable increase in the prevalence of LVD is observed daily. This has led to the identification of a clinical illness, named ischemic cardiomyopathy (ICM) that is characterized by severe coronary artery disease including at least one of the following features: prior revascularization or acute myocardial infarction (AMI); >75% stenosis in the left main stem or the left anterior descending artery; two or more coronary vessels with >75% luminal stenosis.

New devices in the cath lab, such as drug-eluting stents, have been proven superior to obsolete thrombolytic therapy, and more contemporary surgical techniques are associated with improved survival in ICM.

However, among AMI patients, 25–35% will die of sudden cardiac death (SCD) before receiving medical attention, most often from life-threatening tachyarrhythmias such as ventricular tachycardia (VT) and/or ventricular fibrillation (VF) [1,2,3]. While acute in-hospital management of VT/VF is obtained through electrical cardioversion and antiarrhythmic drugs [4,5,6,7], the need for an implantable cardioverter–defibrillator (ICD) in secondary prevention is recommended [8,9,10,11], as well as in primary prevention for patients at risk of malignant ventricular arrhythmias that occur late after discharge.

The evidence is conflicting regarding the association of revascularization and recurrent VT/VF in patients with ICM, with some trials [12] and guidelines [1,13] reporting beneficial association and others reporting no association [14,15,16]. In fact, some patients do not qualify for an ICD according to current guidelines, due to the results of two studies [15,17], which did not show any benefits of early (<40 days after a MI) ICD implantation [16,17,18,19]. New indications are available to guide implantation of an ICD through invasive electrophysiological study (PES, Table 1) for patients with recent MI developing syncope or non-sustained VT, and for patients with indication for a permanent pacemaker due to bradyarrhythmias following MI, who also meet primary prevention criteria for SCD [13,14,15].

On the other hand, the DAPA trial demonstrated that ICD reduces the risk of all-cause mortality at 10 years after implant in patients who suffered a MI [31] and larger studies are ongoing [32].

In light of the above reported considerations, the main goal of treatment for patients dramatically experiencing either ACS or CCS is represented by revascularization, timely performed by PCI (or thrombolysis if PCI is unfeasible) or CABG in cases of symptoms refractory to medical therapy, hemodynamic instability, left main or triple vessel disease, complex coronary anatomy not suitable for PCI, ongoing ischemia despite attempts at PCI or failed PCI [20,33], followed by close clinical re-assessments for establishing ICD indication.

The aim of this review will be to describe the important role of revascularization in favoring positive cardiac remodeling and in preventing VT/VF, as well as the main studies on percutaneous and surgical revascularizations that inspired recommendations of the current guidelines [13,14,15] in seeking complete revascularization followed by further assessments of cardiac function, with conclusive insights on subcutaneous ICD and wearable cardioverter defibrillator implications, as well as the feasibility of remote monitoring of the implanted device when revascularization is incomplete or the patients are yet at risk of SCD.

## 2. Pathophysiologic Benefits of Revascularization

The pathophysiologic substrate of ICM spans from metabolic to neurohumoral and inflammatory changes resulting in an adverse myocardial remodeling and contractile dysfunction in the context of significantly impaired myocardial blood flow and/or reduced coronary flow reserve. Following MI, the onset of myocardial ischemia triggers cellular injury and death, which activate an inflammatory response through inflammasomes, complement activation, and reactive oxygen species production.

Even in the context of successful mechanical revascularization and blood flow restoration in epicardial vessels after AMI, approximately 50% patients do not achieve optimal myocardial reperfusion. The so called no-reflow phenomenon is mainly attributed to coronary microvascular dysfunction that includes impaired vasomotor function, extensive microvascular injury with microvascular obstruction, inflammation and hemorrhage following AMI and reperfusion therapy. Furthermore, the ischemic myocardium undergoes structural and functional remodeling due to stunned and hibernating states [34]. Stunning is referred to the myocardium that is transiently ischemic and displays contractile dysfunction, which ultimately recovers early after restoration of normal resting blood flow. In the context of hibernating myocardium resting blood flow is severely reduced, while cardiac tissue remains viable by adaptively reducing contractility and cellular activity to decrease basal metabolic requests [35]. In contrast to stunned myocardium, hibernating myocardium usually recovers late after revascularization [36]. This implies that an early evaluation after revascularization may underestimate the degree of eventual functional recovery.

However, not all viable myocardium recovers after revascularization and the probability of recovery and reverse remodeling is affected by several factors including the timeliness, completeness, and long-term patency of revascularization. Prolonged myocardial hibernation may progress to necrosis, limiting functional recovery after revascularization. Extent of viability is also important, and several studies have shown that at least 25% to 30% of dysfunctional myocardium needs to be viable for improvement in LVEF after revascularization, with the except of extensively remodeled and dilated ventricles which could not recover after revascularization even in the presence of viable areas [34,35,36].

Interestingly, progress in the diagnosis of myocardial ischemia and the availability of tests to investigate coronary flow reserve and coronary vasomotion in recent years has allowed for better identification and characterization of patients with microvascular angina in everyday clinical practice [37].

Indeed, in the environment of successful mechanical revascularization, previous studies have suggested that low operator and institutional procedures’ volume may be associated with a higher risk of adverse events regarding PCI [38]. The American guidelines and the Society for Cardiovascular Angiography and Intervention [39] recommend that at least 50 coronary interventions must be performed per year (an average of two years) to maintain competency (level of evidence C). In the United Kingdom, the British Cardiovascular Interventional Society believes that independent operators should perform a total of 150 procedures over two years to sustain proficiency [40]. Similar recommendations (class IIa; level of evidence C) are provided in the European Society of Cardiology guidelines for PCI in ACS [41]. The current literature on the relationship between surgical volume and outcomes after PCI provides conflicting data. Some studies reported an increase in adverse event rates due to risk-adjusted operator reduction, whereas other studies did not find this association [42,43,44,45].

Once severe myocardial damage occurs, acute ischemia must be treated as early as possible, and optimal medical therapy (OMT) should significantly reduce morbidity and mortality. [46]. Recently, European guidelines have introduced important innovations on the topic. Specifically, in HF with reduced EF, OMT is based on four basic pillars: angiotensin-converting enzyme inhibitor/type 1—angiotensin II receptor blockers or Sacubitril/Valsartan; sodium-glucose cotransporter-2 inhibitors; beta-adrenergic receptor blockers and mineralocorticoid receptor antagonists. These drugs are strongly recommended to reduce the risk of hospitalization and death [15,47]. Furthermore, OMT has been demonstrated to ameliorate or delay the progression of cardiovascular disease and reduce the annual incidence of SCD [48,49], but it cannot prevent or treat arrhythmic events when they occur. In the analysis of the REVIVED-BCIS2 trial, a strategy of PCI in addition to OMT was not associated with a reduction in all-cause mortality or SCD in patients with ischemic LVD [20]. Despite modern therapies, many patients with reduced LVEF after acute MI are at high risk of SCD, especially during the first months after the infarction [50].

**Figure 1:**
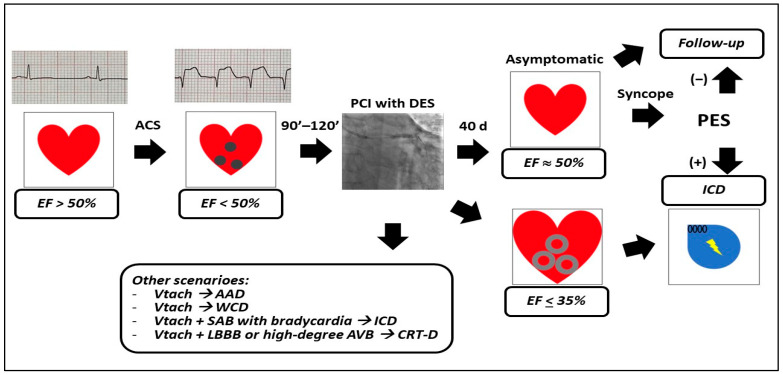
Schematic representation of current guidelines recommendations for sudden cardiac death primary prevention in left ventricular dysfunction after acute coronary syndromes. The spheres represent acute myocardial injury; the circles represent myocardial scars. List of abbreviations: EF: ejection fraction; ACS: acute coronary syndromes; PCI: percutaneous coronary interventions; DES: drug-eluting stents; d: days; PES: programmed electrophysiological study; Vtach: ventricular tachycardia; AAD: antiarrhythmic drugs; WCD: wearable cardioverter defibrillator; SAB: sinoatrial block; ICD: implantable cardioverter defibrillator; LBBB: left bundle branch block; AVB: atrioventricular block; CRT-D: cardiac resynchronization therapy-defibrillator.

**Figure 2:**
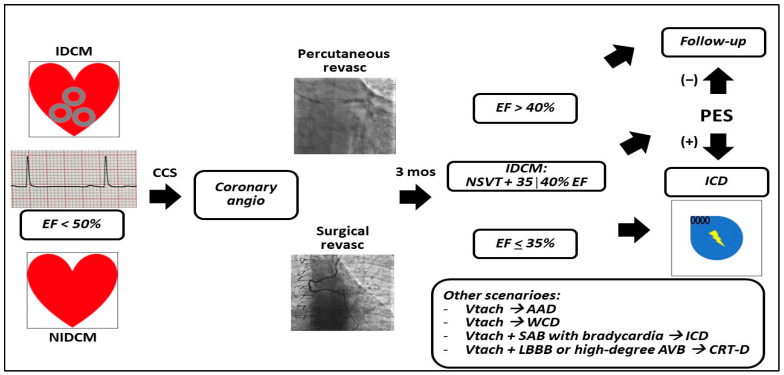
Schematic representation of current guidelines recommendations for sudden cardiac death primary prevention in left ventricular dysfunction in the setting of chronic coronary syndromes. The circles represent myocardial scars. List of abbreviations: IDCM: ischemic dilated cardiomyopathy; NIDCM: non-ischemic dilated cardiomyopathy; EF: ejection fraction; CCS: chronic coronary syndromes; revasc: revascularization; mos: months; PES: programmed electrophysiological study; NSVT: non-sustained ventricular tachycardia; Vtach: ventricular tachycardia; AAD: antiarrhythmic drugs; WCD: wearable cardioverter defibrillator; SAB: sino-atrial block; ICD: implantable cardioverter defibrillator; LBBB: left bundle branch block; AVB: atrioventricular block; CRT-D: cardiac resynchronization therapy-defibrillator.

Therefore, current guidelines [15,16,18] (Figure 1 and Figure 2) recommend routine implantation of ICD for primary prevention of SCD in patients with EF ≤ 35%. 

There is a wide area of scientific investigations about strategies to optimize the current selection criteria for ICD and cardiac resynchronization therapy (CRT) as well [21] (Table 1). Among the recent reports, cardiac sympathetic hyperactivity has been linked to fatal arrhythmias [7] and to response to CRT that can be non-invasively assessed with 123I-meta-iodobenzylguanidine (123I-mIBG) scintigraphy [51]. Another promising tool has been represented by an approach of precision medicine that can offer ICD to patients at a higher range of EF [52].

## 3. Percutaneous Revascularization and ICD Implantation

In the context of a German registry, the beneficial effects of revascularization, mainly percutaneous (74%), have been addressed by serial echocardiography aimed at LVEF quantification 441 ± 220 days following MI. Although a significant increase (more than 6%) was observed, the study shows that several echocardiographic assessments are required before establishing and indication to ICD, in part because of high interindividual as well as intraindividual variability, but mostly because recovery might be delayed by the extension of scar with variable response to therapies and interventions [53].

The REVIVED-BCIS2 prospective, multicenter, open-label, randomized controlled trial [20,54], was originally created for impacting on both revascularization and HF guidelines by demonstrating the role of PCI in the setting of ICM. While the study was designed to address optimal medical therapy alone or in combination with PCI in 700 patients with ICM and viable myocardium, it appeared that in a 3.4 years median of observation, quality of life and cardiac function improved similarly in both groups.

The final superiority data in terms of ICD discharges and lifesaving delivered electrical therapies came from an American registry [55], conducted in consecutive patients hospitalized for MI. More than 11,000 patients, mainly undergone PCI (86%) received in 10% cases an ICD in primary prevention of SCD within 1 year of revascularization. Surprisingly, compared with not implanted patients, those who received an ICD were younger males, and such approach was associated with significantly lower 2-year mortality among revascularized MI patients, mostly upon PCI (Figure 1; Table 2).

## 4. Surgical Revascularization and ICD Implantation

Surgical revascularization has recently been shown to improve long-term outcomes, although the open-chest approach is associated with increased early hazard [58]. A network meta-analysis [59] of on-pump cardioplegic arrest, off-pump, and on-pump beating heart strategies, demonstrated a superiority of the last two approaches in terms of 30-day mortality; such conclusions indicated that critically ill subjects may benefit most from avoiding further myocardial injury associated with cardiopulmonary bypass and cardioplegic arrest. After excluding re-exploration for bleeding or tamponade, patients affected by ICM undergoing CABG surgery show continuous recovery from LVD in the first post-operative year [22,60]. The STICH trial [18] found the highest risk of SCD in the first and in the third months after surgical revascularization in HF patients. Notwithstanding such results, ICD after CABG remains a critical path for patients, physicians, and payers; in fact, yet need for prevention with ICD implantation remains debated due to mortality related to other factors which negatively influence Medicare centers and insurance companies for hospital reimbursement [23,24] (Table 1; Figure 2). Further evidence is therefore required about pre- and post-operative assessments, possibly including surgical repair of the damaged left ventricle, and empowered myocardial imaging that can guide selective surgery.

## 5. ICD Implantation in Ischemic Untreated LVD

Current evidence-based therapy for patients suffering from ICM aims to optimize cardiovascular function, prevent progressive remodeling, reduce HF symptoms and hospitalization, and improve survival. In addition to ICD, CRT and the use of percutaneous device for curing valvular abnormalities such as mitral regurgitation, are recommended treatments when quadruple therapy is already initiated and quintuple therapy by adding vericiguat is considered [25,26,27,56,57,61,62] (Table 2; Figure 1). Combined therapy works along with revascularization, in spite few reports have addressed the worst scenario of no revascularization. In this regard, Gupta and coworkers [63] addressed the association of myocardial ischemia with VT/VF in stable patients with LVD according to stress/rest positron emission tomography (PET) and resting LVEF < 35%. Primary outcome was time-to-first major arrhythmic event, including SCD, resuscitated SCD, or appropriate ICD shocks for VT/VF. A specific subgroup of the study included 365 patients without revascularization after the PET scan.

Takano and coworkers [64] investigated the impact of complete vs. partial revascularization on cardiac dimension and function. The authors studied 56 patients, with more than 80% of cases in which the incompletely revascularized arteries were affecting the LV performance. They observed that significantly greater LV end-diastolic diameter, lower LVEF, and higher prevalence of incomplete revascularization were independent predictors of recurrent VT/VF and appropriate intervention by the implanted ICD compared to other ICD recipients with coronary artery disease and complete revascularization.

Alkharaza and colleagues [28] conducted a large cohort study, observing that a history of revascularization was associated with arrhythmic SCD reduction in patients with a secondary, but not with a primary prevention indication for ICD. Such finding indicates that patients in whom an arrhythmic substrate is found, benefit more from revascularization, and in fact there was major use of amiodarone and digoxin in non-revascularized patients.

However, the largest cohort without revascularization (N = 2855 cases) was addressed in the ACTION Registry–GWTG [55]. This study has indeed the merit of indicating that among more than ten thousand Medicare-insured patients ≥65 years old with reduced EF after MI, clinical follow ups including cardiac function reassessments were useful in patients who were revascularized at the time of MI instead of non-revascularized patients. Therefore, in the ICM setting, revascularization, post-discharge EF re-assessment, clinical follow-ups, and eventual ICD implantation all together represent the standard of medical therapy [29,65,66].

## 6. Impact of COVID-19 Pandemic on Treatment Delay and ICD Implantation

The Coronavirus disease of 2019 (COVID-19), caused by severe acute respiratory syndrome coronavirus 2 (SARS-CoV-2), was declared by the World Health Organization (WHO) on 11 March 2020, was declared a pandemic, and it resulted in a public health crisis of global proportions [67].

In this period, the access to medical care was strongly limited and resulted in a higher rate of medical discontinuation. Additionally, a decline in the emergency department visits and an increase in out-of-hospital CV mortality have been reported. Reorganization of hospitals, focused primarily on managing COVID, was necessary and may lead to neglect of other diseases. To preserve resources, including personal protective equipment and hospital beds to treat COVID-19 patients, the Centers for Disease Control and Prevention has recommended postponing elective cardiac procedures, including coronary angiography and percutaneous coronary intervention, in patients with stable coronary artery disease. In addition, the pandemic reduced medical and paramedical staff available for cardiac disease for prioritize care for COVID patients [68,69,70].

Several works demonstrated the dramatic reduction in PCI, even in ACS patients [71,72]. One trial conducted during the early phase of the COVID pandemic in the U.S. showed an estimated 38% reduction in ST-elevation MI activations cardiac cath lab [73]. Another study confirmed the significant difference in the admission to intensive care unit for all causes and ACS, between the pre-lockdown and post-lockdown periods, with a rebound after lockdown. Conversely, length-of-stay and in-hospital mortality did not show any change from to pre-pandemic in the entire population [74].

COVID-19 had also a significant influence on cardiac arrhythmia care, resulting in a greater than 50% decrease in all types of operations [75]. The time from symptoms to the first medical contact was shorter for arrhythmic emergencies requiring a device implant, but the time from the initial medical contact to the procedure was much longer. The number of elective pacemaker (PM), ICD, and CRT devices implantations decreased by 40% compared to 2019 and by 70% for ablations during the first wave. Subsequent waves typically saw a slow return to pre-pandemic levels for ablations. Recovery after the initial 10% reduction for emergency procedures (PM, ICD, CRT, and ablations) happened usually during the second wave, with some variation [76,77].

Remarkable, pandemic’s disruption of healthcare provision had a significant and pro-active impact on the widespread adoption of telemedicine, which helped to address the challenges of providing care for chronic diseases using the traditional approach based on in-person visits and checks. The more widespread use of telemedicine should be promoted to reduce the risk of infection, to maintain continuity of care and prompt optimization of medical therapy, given the limitations to normal care and high morbidity and death among HF patients developing COVID-19. During the COVID-19 epidemic, this technology has been used successfully to give medical advice, alter medication, and monitor ambulatory HF patients. [78,79]

## 7. Emerging Technologies for Short- and Long-Term Management of SCD Risk in Ischemic Cardiomyopathy

The frontiers in SCD prevention are directed toward expanding indications while reducing risks, which both are related to the role of ICD in the young patients, as well as in the acute setting. Younger subjects are exposed to a potential risk over the longer follow-up compared to middle-aged patients, since they will undergo several generator replacements. The subcutaneous ICD (S-ICD), besides its wide adoption in the above-described setting [80], is a valid therapy also in ICM, since it does not enter the thorax or pleural space, and therefore limits the risk of bleeding that derives from vein access, and pocket hematoma. As S-ICD is highly reliable in detecting and treating VT/VF by using a shock-lead that is located subcutaneously above the sternum, the increased reliability of the surface leads is encountered also in the wearable cardioverter–defibrillator (WCD, Table 3; Figure 1 and Figure 2).

Currently, such technology is offered to patients at high risk for SCD not fulfilling criteria for ICD implantation, mainly because close temporal relationship with either MI or revascularization [81,82,83,84,85]. While the WEARIT and BIROAD studies [86,87] addressed the feasibility of VT/VF detection and treatment by using the WCD, the WEARIT II [88] and the recent VEST trials [89] provided conflicting results in terms of safely and effectively preventing SCD in the first period after MI in patients with LVD. Unfortunately, besides optimal medical therapy, it remains unclear how to reduce the risk of SCD in the early period after MI, that is when ICDs are contraindicated.

## 8. Conclusions and Future Perspectives

A full restoration of cardiac function back to the frame preceding the acute MI is improbable to obtain. Although complete revascularization is mandatory for positive ventricular remodeling and reduction in arrhythmic burden, the actual recovery is modest in patients with either severely reduced LVEF or with moderate reduction. Stunned and hibernated myocardium are the clinical scenarios that benefit the most from revascularization. On the other hand, recently introduced “pillars” of heart failure therapy, when initiated timely, contribute dramatically to the outcomes of HF patients.

While ICD has demonstrated a superiority in terms of lives saved [30,90], it is also important to assess whether revascularization, obtained either through PCI or CABG, is complete in order to achieve improved cardiac performances and outcome, similarly to reduction in fatal arrhythmias in the ischemic and post-ischemic settings. The role of revascularization yet remains controversial, mostly due to the selected approach based either on optimal medical therapy alone or in combination with routine angiography and revascularization [91,92].

Amelioration in LVEF due to medical therapy, revascularization, CRT, and transcatheter edge-to-edge repair of the mitral regurgitation could lead to improvement in prognosis and may modify the association of myocardial scar and ischemia-related outcomes [21,51].

Finally, remote monitoring has also shown a reduction in severe outcomes when applied to loop recorders inserted in ischemic patients [93], as well as in HF patients implanted with ICD for reducing inappropriate shocks [94]. Further research will hopefully clarify which patient requires primary prevention with ICD independently by revascularization, as well as the clinical setting in which a complete revascularization can be considered sufficient to terminate the intrinsic arrhythmic risk of the injured myocardium.

## Figures and Tables

**Table 1:** Randomized trials of ICD therapy in coronary artery disease.

Study	Main Findings
AlTurki, A. et al. [3]	ICD therapy is superior to antiarrhythmic-drug therapy
Mittal, S. et al. [5]	One third of patients with non-sustained VT during the early post-revascularization period and with inducible VT/VF at PES have a high incidence of arrhythmic events and receive appropriate ICD therapy
Buxton, A.E. et al. [6]	Asymptomatic patients with coronary artery disease, LV dysfunction, non-sustained VT and negative PES have a significantly lower risk of SCD and lower overall mortality than similar patients with inducible sustained VT
Raitt, M.H. et al. [8]	ICD implantation in ICM aggravated by sustained VT reduces mortality
Connolly, S.J. et al. [9]	ICD is superior to amiodarone in reducing all-cause mortality
Kuck, K.-H. et al. [10]	ICD reduces all-cause mortality compared to amiodarone/metoprolol, mostly during the first 5 years after the index event
Rao, M.P. et al. [18]	Risk stratification in ICD recipients after CABG and reduced LVEF is mandatory
Perera, D. et al. [20]	PCI not reduced SCD and VT/VF. In patients who are eligible for an ICD, implantation does not need to be deferred
Theuns, D.A.M.J. et al. [21]	ICD improves survival in ischemic or non-ischemic heart disease with LVEF ≤ 35% when implanted after either 40 days from MI or ≥3 months from coronary revascularization
Vakil, K. et al. [22]	Mild improvement in severely depressed (<25% LVEF) patients before surgical revascularization
Cook, J.R. et al. [23]	Revascularization and ICD improve survival in out-of-hospital cardiac arrest
Smith, T. et al. [24]	Cost-effective analysis of SCD primary prevention in patients with LVEF < 40% and ischemic or non-ischemic heart disease shows superiority of ICD
Cantero-Pérez, E. et al. [25]	ICD reduces the risk of SCD in patients with LVEF ≤ 30% while awaiting HTx
Fröhlich, G.M. et al. [26]	ICD provides an immediate and sustained survival benefit for patients awaiting HTx
Gandjbakhch, E. et al. [27]	ICD has a little benefit on survival in overt HF patients awaiting HTx
Alkharaza, A. et al. [28]	Therapy with ICD improves survival on the HTx wait list independently by LVAD
Looi, K.-L. et al. [29]	ICD reduces mortality in HF patients with NYHA class II or III and ≤35% LVEF compared to amiodarone
Raphael, C.E. et al. [30]	ICD in primary prevention lowers patients’ risk over their lifetime

List of abbreviations: ICD: implantable cardioverter–defibrillator; VT: ventricular tachycardia; VF: ventricular fibrillation; PES: programmed electrophysiological study; LV: left Ventricle; SCD: sudden cardiac death; ICM: ischemic cardiomyopathy; CABG: coronary artery bypass grafting; LVEF: left ventricular ejection fraction; MI: myocardial infarction; HTx: heart transplantation; HF: heart failure; LVAD: left ventricular assist devices; NYHA: New York Heart Association.

**Table 2:** Randomized trials of ICD therapy in acute myocardial infarction.

Study	Main Findings
Pauriah, M. et al. [4]	A stepwise approach with ablation as first-line treatment in post-infarct patients presenting with VT might be a reasonable option
Kuck, K.-H. et al. [7]	Prophylactic VT ablation before ICD delays arrhythmia relapse in patients with stable VT, previous MI, and reduced LVEF
Hohnloser, S.H. et al. [17]	Prophylactic ICD therapy does not reduce overall mortality in high-risk patients with re-cent MI due to an increase in the rate of non-arrhythmic death
Steinbeck, G. et al. [16]	Prophylactic ICD therapy does not reduce overall mortality in MI patients with clinical features that increase risk
Moss, A.J. et al. [19]	Prophylactic implantation of a defibrillator improves survival
Haanschoten, D.M. et al. [31]	Prophylactic ICD improves survival as compared with conventional medical therapy in patients with high-risk MI
Zaman, S. et al. [32]	Early ICD implantation only for patients with impaired LVEF and inducible VT after primary PCI following ST-elevation MI
Goldstein, S.A. et al. [55]	ICD implantation was associated with lower mortality following revascularization for MI
Greenberg, H. et al. [56]	Significant SCD reduction (10.0%) after ICD compared to conventional group (3.8%) in the ICD group
Sesselberg, H.W. et al. [57]	Higher mortality in MADIT-II patients with severely reduced LVEF and ES after MI compared to post-infarction with only isolated VT/VF as well as those without any episodes of VT/VF

List of abbreviations: ICD: implantable cardioverter–defibrillator; VT: ventricular tachycardia; MI: myocardial infarction; LVEF: left ventricular ejection fraction; PCI: percutaneous coronary interventions; SCD: sudden cardiac death; MADIT: Multicenter Automatic Defibrillator Implantation Trial; ES: electrical storm; VF: ventricular fibrillation.

**Table 3:** Randomized trials of wearable cardioverter defibrillator therapy in acute myocardial infarction.

Study	Main Findings
Zishiri, E.T. et al. [81]	Proof-of-concept study for WCD use as a bridge therapy after coronary revascularization
Epstein, A.E. et al. [82]	The study showed the beneficial effect of WCD during the first month of use
Cheung, C.C. et al. [83]	WCD leads to a modest reduction in arrhythmic death but a nominally significant reduction in all-cause mortality
Adler, A. et al. [84]	WCD requires patients capable of using it who are at high risk of VT/VF; it can also be considered as a long-term solution in unique cases with contraindications for an ICD
Garcia, R. et al. [85]	WCD works better when its use is preceded by patient education accompanied by dedicated remote monitoring follow-up
Kondo, Y. et al. [86]	A time limited WCD therapy prevents SCD in the acute setting after MI
Hioki, H. et al. [87]	WCD recognizes and treats appropriately VTs in patients not qualifying for an ICD, and is a useful bridge to HTx
Kutyifa, V. et al. [88]	Patients who do not qualify for ICD have high rate of sustained VT at 3 months
Olgin, J.E. et al. [89]	WCD does not reduce rate of arrhythmic death compared to control in patients with a recent MI and LVEF ≤ 35%

WCD: wearable cardioverter defibrillator; VT: ventricular tachycardia; VF: ventricular fibrillation; ICD: implantable cardioverter–defibrillator; SCD: sudden cardiac death; MI: myocardial infarction; HTx: heart transplantation; LVEF: Left ventricular ejection fraction.

## Data Availability

Not applicable.

## References

[1] Nageh M.F., Kim J.J., Chen L.-H., Yao J.F. (2014). Implantable Defibrillators for Secondary Prevention of Sudden Cardiac Death in Cardiac Surgery Patients with Perioperative Ventricular Arrhythmias. J. Am. Heart Assoc..

[2] Madhavan M., Friedman P.A., Lennon R.J., Prasad A., White R.D., Sriram C.S., Gulati R., Gersh B.J. (2015). Implantable cardioverter-defibrillator therapy in patients with ventricular fibrillation out of hospital cardiac arrest secondary to acute coronary syndrome. J. Am. Heart Assoc..

[3] AlTurki A., Proietti R., Russo V., Dhanjal T., Banerjee P., Essebag V. (2019). Anti-arrhythmic drug therapy in implantable cardioverter-defibrillator recipients. Pharmacol. Res..

[4] Pauriah M., Cismaru G., Magnin-Poull I., Andronache M., Sellal J.-M., Schwartz J., Brembilla-Perrot B., Sadoul N., Aliot E., de Chillou C. (2013). A Stepwise Approach to the Management of Postinfarct Ventricular Tachycardia Using Catheter Ablation as the First-Line Treatment. A Single-Center Experience. Circ. Arrhythmia Electrophysiol..

[5] Mittal S., Lomnitz D.J., Mirchandani S., Stein K.M., Markowitz S.M., Slotwiner D.J., Iwai S., Das M.K., Lerman B.B. (2002). Prognostic significance of nonsustained ventricular tachycardia after revascularization. J. Cardiovasc. Electrophysiol..

[6] Buxton A.E., Lee K.L., DiCarlo L., Gold M.R., Greer G.S., Prystowsky E.N., O’Toole M.F., Tang A., Fisher J.D., Coromilas J. (2000). Electrophysiologic testing to identify patients with coronary artery disease who are at risk for sudden death. N. Engl. J. Med..

[7] Kuck K.-H., Schaumann A., Eckardt L., Willems S., Ventura R., Delacrétaz E., Pitschner H.-F., Kautzner J., Schumacher B., Hansen P.S. (2010). Catheter ablation of stable ventricular tachycardia before defibrillator implantation in patients with coronary heart disease (VTACH): A multicentre randomised controlled trial. Lancet.

[8] Raitt M.H., Renfroe E.G., Epstein A.E., McAnulty J.H., Mounsey P., Steinberg J.S., Lancaster S.E., Jadonath R.L., Hallstrom A.P. (2001). “Stable” Ventricular Tachycardia Is Not a Benign Rhythm. Insights From the Antiarrhythmics Versus Implantable Defibrillators (AVID) Registry. Circulation.

[9] Connolly S.J., Gent M., Roberts R.S., Dorian P., Roy D., Sheldon R.S., Mitchell L.B., Green M.S., Klein G.J., O’Brien B. (2000). Canadian Implantable Defibrillator Study (CIDS). A Randomized Trial of the Implantable Cardioverter Defibrillator Against Amiodarone. Circulation.

[10] Kuck K.-H., Cappato R., Siebels J., Rüppel R. (2000). Randomized Comparison of Antiarrhythmic Drug Therapy with Implantable Defibrillators in Patients Resuscitated from Cardiac Arrest. The Cardiac Arrest Study Hamburg (CASH). Circulation.

[11] Connolly S.J., Hallstrom A.P., Cappato R., Schron E.B., Kuck K.-H., Zipes D.P., Greene H.L., Boczor S., Domanski M., Follmann D. (2000). Meta-analysis of the implantable cardioverter defibrillator secondary prevention trials. Eur. Heart J..

[12] Liang J.J., Hodge D.O., Mehta R.A., Russo A.M., Prasad A., Cha Y.-M. (2014). Outcomes in patients with sustained ventricular tachyarrhythmias occurring within 48 h of acute myocardial infarction: When is ICD appropriate?. Europace.

[13] Zeppenfeld K., Tfelt-Hansen J., de Riva M., Winkel B.G., Behr E.R., Blom N.A., Charron P., Corrado D., Dagres N., de Chillou C. (2022). ESC Scientific Document Group, 2022 ESC Guidelines for the management of patients with ventricular arrhythmias and the prevention of sudden cardiac death: Developed by the task force for the management of patients with ventricular arrhythmias and the prevention of sudden cardiac death of the European Society of Cardiology (ESC) Endorsed by the Association for European Paediatric and Congenital Cardiology (AEPC). Eur. Heart J..

[14] Al-Khatib S.M., Stevenson W.G., Ackerman M.J., Bryant W.J., Callans D.J., Curtis A.B., Deal B.J., Dickfeld T., Field M.E., Fonarow G.C. (2018). 2017 AHA/ACC/HRS Guideline for Management of Patients with Ventricular Arrhythmias and the Prevention of Sudden Cardiac Death; A Report of the American College of Cardiology/American Heart Association Task Force on Clinical Practice Guidelines and the Heart Rhythm Society. Circulation.

[15] McDonagh T.A., Metra M., Adamo M., Gardner R.S., Baumbach A., Böhm M., Burri H., Butler J., Čelutkienė J., Chioncel O. (2021). ESC Scientific Document Group 2021 ESC Guidelines for the diagnosis and treatment of acute and chronic heart failure: Developed by the Task Force for the diagnosis and treatment of acute and chronic heart failure of the European Society of Cardiology (ESC) With the special contribution of the Heart Failure Association (HFA) of the ESC. Eur. Heart J..

[16] Steinbeck G., Andresen D., Seidl K., Brachmann J., Hoffmann E., Wojciechowski D., Kornacewicz-Jach Z., Sredniawa B., Lupkovics G., Hofgärtner F. (2009). Defibrillator Implantation Early After Myocardial Infarction. N. Engl. J. Med..

[17] Hohnloser S.H., Kuck K.H., Dorian P., Roberts R.S., Hampton J.R., Hatala R., Fain E., Gent M., Connolly S.J. (2004). Prophylactic use of an implantable cardioverter-defibrillator after acute myocardial infarction. N. Engl. J. Med..

[18] Rao M.P., Al-Khatib S.M., Pokorney S.D., She L., Romanov A., Nicolau J.C., Lee K.L., Carson P., Selzman C.H., Stepinska J. (2017). Sudden Cardiac Death in Patients with Ischemic Heart Failure Undergoing Coronary Artery Bypass Grafting: Results from the STICH Randomized Clinical Trial (Surgical Treatment for Ischemic Heart Failure). Circulation.

[19] Moss A.J., Zareba W., Hall W.J., Klein H., Wilber D.J., Cannom D.S., Daubert J.P., Higgins S.L., Brown M.W., Andrews M.L. (2002). Prophylactic implantation of a defibrillator in patients with myocardial infarction and reduced ejection fraction. N. Engl. J. Med..

[20] Perera D., Morgan H.P., Ryan M., Dodd M., Clayton T., O’Kane P.D., Greenwood J.P., Walsh S.J., Weerackody R., McDiarmid A. (2023). Arrhythmia and Death Following Percutaneous Revascularization in Ischemic Left Ventricular Dysfunction: Prespecified Analyses From the REVIVED-BCIS2 Trial. Circulation.

[21] Theuns D.A.M.J., Smith T., Hunink M.G.M., Bardy G.H., Jordaens L. (2010). Effectiveness of prophylactic implantation of cardioverter-defibrillators without cardiac resynchronization therapy in patients with ischaemic or non-ischaemic heart disease: A systematic review and meta-analysis. Europace.

[22] Vakil K., Florea V., Koene R., Kealhofer J.V., Anand I., Adabag S. (2016). Effect of coronary artery bypass grafting on left ventricular ejection fraction in men eligible for implantable cardioverter-defibrillator. Am. J. Cardiol..

[23] Cook J.R., Rizo-Patron C., Curtis A.B., Gillis A.M., Bigger J.T., Kutalek S.P., Coromilas J., Hofer B.I., Powell J., Hallstrom A.P. (2002). Effect of surgical revascularization in patients with coronary artery disease and ventricular tachycardia or fibrillation in the Antiarrhythmics Versus Implantable Defibrillators (AVID) Registry. Am. Heart J..

[24] Smith T., Jordaens L., Theuns D.A.M.J., van Dessel P.F., Wilde A.A., Hunink M.G.M. (2013). The cost-effectiveness of primary prophylactic implantable defibrillator therapy in patients with ischaemic or non-ischaemic heart disease: A European analysis. Eur. Heart J..

[25] Cantero-Pérez E.M., Sobrino-Márquez J.M., Grande-Trillo A., Lage-Gallé E., Rangel-Sousa D., Esteve-Ruiz I.M. (2013). A Martínez-Martínez Implantable cardioverter defibrillator for primary prevention in patients with severe ventricular dysfunction awaiting heart transplantation. Transplant. Proc..

[26] Fröhlich G.M., Holzmeister J., Hübler M., Hübler S., Wolfrum M., Enseleit F., Seifert B., Hürlimann D., Lehmkuhl H.B., Noll G. (2013). Prophylactic implantable cardioverter defibrillator treatment in patients with end- stage heart failure awaiting heart transplantation. Heart J..

[27] Gandjbakhch E., Rovan M., Varnous S., Maupai C., Chastre T., Waintraub X., Pousset F., Lebreton G., Duthoit G., Badenco N. (2016). Implantable cardioverter-defibrillators in end-stage heart failure patients listed for heart transplantation: Results from a large retrospective registry. Arch. Cardiovasc..

[28] Alkharaza A., Al-Harbi M., El-Sokkari I., Doucette S., MacIntyre C., Gray C., Abdelwahab A., Sapp J.L., Gardner M., Parkash R. (2020). The effect of revascularization on mortality and risk of ventricular arrhythmia in patients with ischemic cardiomyopathy. BMC Cardiovasc. Disord..

[29] Looi K.-L., Lever N., Tang A., Agarwal S. (2017). Prophylactic implantable cardioverter defibrillator in heart failure: The growing evidence for all or Primum non nocere for some?. Heart Fail. Rev..

[30] Raphael C.E., Finegold J.A., Barron A.J., Whinnett Z.I., Mayet J., Linde C., Cleland J.G.F., Levy W.C., Francis D.P. (2015). The effect of duration of follow-up and presence of competing risk on lifespan-gain from implantable cardioverter defibrillator therapy: Who benefits the most?. Eur. Heart J..

[31] Haanschoten D.M., Elvan A., Ramdat Misier A.R., Delnoy P.P.H.M., Smit J.J.J., Adiyaman A., Demirel F., Wellens H.J.J., Verheugt F.W.A., Ottervanger J.P. (2020). Long-Term Outcome of the Randomized DAPA Trial. Circ. Arrhythm. Electrophysiol..

[32] Zaman S., Sivagangabalan G., Narayan A., Thiagalingam A., Ross D.L., Kovoor P. (2009). Outcomes of early risk stratification and targeted implantable cardioverter-defibrillator implantation after ST-elevation myocardial infarction treated with primary percutaneous coronary intervention. Circulation.

[33] Ali Z.A., Horst J., Gaba P., Shaw L.J., Bangalore S., Hochman J.S., Maron D.J., Moses J.W., Alfonso M.A., Madhavan M.V. (2021). Standardizing the Definition and Analysis Methodology for Complete Coronary Artery Revascularization. J. Am. Heart Assoc..

[34] Cousoumbas G.V., Casella G., Di Pasquale G. (2023). What is the role of coronary revascularization to recover the contractility of the dysfunctional heart?. Eur. Heart J. Suppl..

[35] Katikireddy C.K., Samim A. (2022). Myocardial viability assessment and utility in contemporary management of ischemic cardiomyopathy. Clin. Cardiol..

[36] Parikh K., Choy-Shan A., Ghesani M., Donnino R. (2021). Multimodality Imaging of Myocardial Viability. Curr. Cardiol. Rep..

[37] Kaski J.-C., Crea F., Gersh B.J., Camici P.G. (2018). Reappraisal of Ischemic Heart Disease- Fundamental Role of Coronary Microvascular Dysfunction in the Pathogenesis of Angina Pectoris. Circulation.

[38] Zabojszcz M., Januszek R., Siudak Z., Janion-Sadowska A., Jędrychowska M., Pawlik A., Tokarek T., Staszczak B., Malinowski K.P., Bartuś S. (2020). Association between the mortality rate and operator volume in patients undergoing emergency or elective percutaneous coronary interventions. Kardiol. Pol..

[39] Nallamothu B.K., Tommaso C.L., Anderson H.V., Anderson J.L., Cleveland J.C., Dudley R.A., Duffy P.L., Faxon D.P., Gurm H.S., Hamilton L.A. (2014). ACC/AHA/SCAI/AMA-Convened PCPI/NCQA 2013 Performance Measures for Adults Undergoing Percutaneous Coronary Intervention: A Report of the American College of Cardiology/American Heart Association Task Force on Performance Measures, the Society for Cardiovascular Angiography and Interventions, the American Medical Association-Convened Physician Consortium for Performance Improvement, and the National Committee for Quality Assurance. Circulation.

[40] Gray H.H. (2000). Coronary angioplasty: Guidelines for good practice and training. Joint working group on coronary angioplasty of the British Cardiac Society and British Cardiovascular Intervention Society. Heart.

[41] Windecker S., Kolh P., Alfonso F., Collet J.-P., Cremer J., Falk V., Filippatos G., Hamm C., Head S.J., Jüni P. (2014). 2014 ESC/EACTS Guidelines on myocardial revascularization: The Task Force on Myocardial Revascularization of the European Society of Cardiology (ESC) and the European Association for Cardio-Thoracic Surgery (EACTS) Developed with the special contribution of the European Association of Percutaneous Cardiovascular Interventions (EAPCI). Eur. Heart J..

[42] Strom J.B., Wimmer N.J., Wasfy J.H., Kennedy K., Robert W., Association Between Operator Procedure Volume and Patient Outcomes in Percutaneous Coronary Intervention (2014). A Systematic Review and Meta-Analysis. Circ. Cardiovasc. Qual. Outcomes.

[43] Badheka A.O., Patel N.J., Grover P., Singh V., Patel N., Arora S., Chothani A., Mehta K., Deshmukh A., Savani G.T. (2014). Impact of Annual Operator and Institutional Volume on Percutaneous Coronary Intervention Outcomes A 5-Year United States Experience (2005–2009). Circulation.

[44] Maroney J., Khan S., Powell W., Klein L.W. (2013). Current operator volumes of invasive coronary procedures in medicare patients: Implications for future manpower needs in the catheterization laboratory. Catheter. Cardiovasc. Interv..

[45] Hulme W., Sperrin M., Curzen N., Kinnaird T., De Belder M.A., Ludman P., Kwok C.S., Gale C.P., Cockburn J., Kontopantelis E. (2018). Operator volume is not associated with mortality following percutaneous coronary intervention: Insights from the British Cardiovascular Intervention Society registry. Eur. Heart J..

[46] Fragasso G. (2023). Severe Left Ventricular Dysfunction After Acute Myocardial Infarction: A Call for Development of Adequately Targeted Treatments. Am. J. Cardiol..

[47] McDonagh T.A., Metra M., Adamo M., Gardner R.S., Baumbach A., Böhm M., Burri H., Butler J., Čelutkienė J., Chioncel O. (2023). 2023 Focused Update of the 2021 ESC Guidelines for the diagnosis and treatment of acute and chronic heart failure: Developed by the task force for the diagnosis and treatment of acute and chronic heart failure of the European Society of Cardiology (ESC) With the special contribution of the Heart Failure Association (HFA) of the ESC. Eur. Heart J..

[48] McMurray J.J., Packer M., Desai A.S., Gong J., Lefkowitz M.P., Rizkala A.R., Rouleau J.L., Shi V.C., Solomon S.D., Swedberg K. (2014). PARADIGM-HF Investigators and Committees. Angiotensin-neprilysin inhibition versus enalapril in heart failure. N. Engl. J. Med..

[49] Shen L., Jhund P.S., Petrie M.C., Claggett B.L., Barlera S., Cleland J.G.F., Dargie H.J., Granger C.B., Kjekshus J., Kuber L. (2017). Declining risk of sudden death in heart failure. N. Engl. J. Med..

[50] Sjöblom J., Muhrbeck J., Witt N., Alam M., Frykman-Kull V. (2014). Evolution of Left Ventricular Ejection Fraction After Acute Myocardial Infarction Implications for Implantable Cardioverter-Defibrillator Eligibility. Circulation.

[51] Curcio A., Cascini G.L., De Rosa S., Pasceri E., Veneziano C., Cipullo S., Saccomanno M., Santarpia G., Cuda G., Indolfi C. (2016). (123)I-mIBG imaging predicts functional improvement and clinical outcome in patients with heart failure and CRT implantation. Int. J. Cardiol..

[52] Dagres N., Peek N., Leclercq C., Hindricks G. (2020). The PROFID project. Eur. Heart J..

[53] Reibis R., Salzwedel A., Bonaventura K., Völler H., Wegscheider K. (2017). Improvement of left ventricular ejection fraction in revascularized postmyocardial patients: Indication for statistical fallacy. BMC Res. Notes.

[54] Perera D., Clayton T., O’Kane P.D., Greenwood J.P., Weerackody R., Ryan M., Morgan H.P., Dodd M., Evans R., Canter R. (2022). Percutaneous Revascularization for Ischemic Left Ventricular Dysfunction. N. Engl. J. Med..

[55] Goldstein S.A., Li S., Lu D., Matsouaka R.A., Rymer J., Fonarow G.C., de Lemos J.A., Peterson E., Pokorney S.D., Wang T. (2021). Implantable cardioverter defibrillator utilization and mortality among patients ≥65 years of age with a low ejection fraction after coronary revascularization. Am. J. Cardiol..

[56] Greenberg H., Case R.B., Moss A.J., Brown M.W., Carroll E.R., Andrews M.L. (2004). MADIT-II Investigators Analysis of mortality events in the Multicenter Automatic Defibrillator Implantation Trial (MADIT-II). J. Am. Coll. Cardiol..

[57] Sesselberg H.W., Moss A.J., McNitt S., Zareba W., Daubert J.P., Andrews M.L., Hall W.J., McClinitic B., Huang D.T. (2007). Ventricular arrhythmia storms in postinfarction patients with implantable defibrillators for primary prevention indications: A MADIT-II substudy. Heart Rhythm J..

[58] Adabag S., Carlson S., Gravely A., Buelt-Gebhardt M., Madjid M., Naksuk N. (2022). Improvement of left ventricular function with surgical revascularization in patients eligible for implantable cardioverter-defibrillator. J. Cardiovasc. Electrophysiol..

[59] Hwang B., Williams M.L., Tian D.H., Yan T.D., Misfeld M. (2022). Coronary artery bypass surgery for acute coronary syndrome: A network meta-analysis of on-pump cardioplegic arrest, off-pump, and on-pump beating heart strategies. J. Card. Surg..

[60] Ntinopoulos V., Papadopoulos N., Odavic D., Haeussler A., Dzemali O. (2022). Ejection Fraction Recovery after Coronary Artery Bypass Grafting for Ischemic Cardiomyopathy. Thorac. Cardiovasc. Surg..

[61] Gupta A., Pasupula D.K., Bhonsale A., Kancharla K., Wang N.C., Adelstein E., Jain S., Saba S. (2018). Implantable cardioverter-defibrillator therapy in device recipients who survived a cardiac arrest associated with a reversible cause. J. Cardiovasc. Electrophysiol..

[62] Vakil K., Duval S., Cogswell R., Eckman P., Levy W.C., Anand I., Dardas T., Adabag S. (2017). Impact of Implantable Cardioverter-Defibrillators on Waitlist Mortality Among Patients Awaiting Heart Transplantation: An UNOS/OPTN Analysis. JACC Clin. Electrophysiol..

[63] Gupta A., Harrington M., Albert C.M., Bajaj N.S., Hainer J., Morgan V., Bibbo C.F., Bravo P.E., Osborne M.T., Dorbala S. (2018). Myocardial scar but not ischemia is associated with defibrillator shocks and sudden cardiac death in stable patients with reduced left ventricular ejection fraction. JACC Clin. Electrophysiol..

[64] Takano T., Tanaka K., Ozaki K., Sato A., Iijima K., Yanagawa T., Izumi D., Ozawa T., Fuse K., Sato M. (2018). Clinical Predictors of Recurrent Ventricular Arrhythmias in Secondary Prevention Implantable Cardioverter Defibrillator Recipients with Coronary Artery Disease—Lower Left Ventricular Ejection Fraction and Incomplete Revascularization. Circ. J..

[65] Pokorney S.D., Miller A.L., Chen A.Y., Thomas L., Fonarow G.C., de Lemos J.A., Al-Khatib S.M., Velazquez E.J., Peterson E.D., Wang T.Y. (2017). Reassessment of cardiac Function and implantable cardioverter-Defibrillator Use among Medicare Patients With low ejection Fraction after Myocardial infarction. Circulation.

[66] Beg F., Valderrabano M., Schurmann P. (2020). Device Therapy for Sudden Cardiac Death Prophylaxis After Acute Coronary Syndrome: When and Why?. Curr. Cardiol. Rep..

[67] Indolfi C., Spaccarotella C. (2020). The Outbreak of COVID-19 in Italy: Fighting the Pandemic. JACC Case Rep..

[68] The Task Force for the Management of COVID-19 of the European Society of Cardiology (2022). ESC guidance for the diagnosis and management of cardiovascular disease during the COVID-19 pandemic: Part 2—Care pathways, treatment, and follow-up. Eur. Heart J..

[69] Palazzuoli A., Metra M., Collins S.P., Adamo M., Ambrosy A.P., Antohi L.E., Ben Gal T., Farmakis D., Gustafsson F., Hill L. (2022). Heart failure during the COVID-19 pandemic: Clinical, diagnostic, management, and organizational dilemmas. ESC Heart Fail..

[70] AHA’s Mission: Lifeline (2020). Temporary Emergency Guidance to STEMI Systems of Care During the COVID-19 Pandemic: AHA’s Mission: Lifeline. Circulation.

[71] Campanile A., Verdecchia P., Ravera A., Coiro S., Mattei C., Scavelli F., Bearzot L., Cutolo A., Centola M., Carugo S. (2021). Intensive cardiac care unit admission trends during the COVID-19 outbreak in Italy: A multi-center study. Intern. Emerg. Med..

[72] De Rosa S., Spaccarotella C., Basso C., Calabrò M.P., Curcio A., Filardi P.P., Mancone M., Mercuro G., Muscoli S., Nodari S. (2020). Reduction of hospitalizations for myocardial infarction in Italy in the COVID-19 era. Eur. Heart J..

[73] Garcia S., Albaghdadi M., Meraj P., Schmidt C., Garberich R., Jaffer F.A., Dixon S., Rode J.J., Tannenbaum M., Chambers J. (2020). Reduction in ST-Segment Elevation Cardiac Catheterization Laboratory Activations in the United States During COVID-19 Pandemic. J. Am. Coll. Cardiol..

[74] Spaccarotella C., Sorrentino S., Indolfi C. (2021). Reduction of hospitalisations and increased mortality for acute coronary syndromes during COVID-19 era: Not all countries are equal. Lancet Reg. Health West Pac..

[75] Menichetti F., Nesti M., Notarstefano P., Fazi A., Del Rosso A., Solarino G., La Pira F., Giorgi D., Arena G., Rossi A. (2021). Prolonged care delivery time and reduced rate of electrophysiological procedures during the lockdown period due to COVID-19 outbreak. Expert Rev. Med. Devices.

[76] Schwab J.O., Wiese J., Hauser T. (2022). The influence of the 2020 COVID-19 pandemic on the implantation rates of cardiac implantable electronic devices in Germany: Changes between 2020 Q1–Q3 and 2019 Q1–Q3. Eur. Heart J. Qual. Care Clin. Outcomes.

[77] Boriani G., Guerra F., De Ponti R., D’Onofrio A., Accogli M., Bertini M., Bisignani G., Forleo G.B., Landolina M., Lavalle C. (2023). Five waves of COVID-19 pandemic in Italy: Results of a national survey evaluating the impact on activities related to arrhythmias, pacing, and electrophysiology promoted by AIAC (Italian Association of Arrhythmology and Cardiac Pacing). Intern. Emerg. Med..

[78] Curcio A., Spaccarotella C., Brunetti N.D., Molinari G., Carugo S., Basso C., Ciccone M.M., Filardi P.P., Mancone M., Mercuro G. (2021). Italian Society of Cardiology (SIC) Position paper: Technical, instrumental and standards of interpretation for electrocardiography, ambulatory electrocardiographic and blood pressure monitoring in telemedicine. G Ital. Cardiol..

[79] Brunetti N.D., Curcio A., Nodari S., Parati G., Carugo S., Molinari M., Acquistapace F., Gensini G., Molinari G. (2023). The Italian Society of Cardiology and Working Group on Telecardiology and Informatics 2023 updated position paper on telemedicine and artificial intelligence in cardiovascular disease. J. Cardiovasc. Med..

[80] Migliore F., Biffi M., Viani S., Pittorru R., Francia P., Pieragnoli P., De Filippo P., Bisignani G., Nigro G., Dello Russo A. (2023). Modern subcutaneous implantable defibrillator therapy in patients with cardiomyopathies and channelopathies: Data from a large multicentre registry. Europace.

[81] Zishiri E.T., Williams S., Cronin E.M., Blackstone E.H., Ellis S.G., Roselli E.E., Smedira N.G., Gillinov A.M., Glad J.A., Tchou P.J. (2013). Early risk of mortality after coronary artery revascularization in patients with left ventricular dysfunction and potential role of the wearable cardioverter defibrillator. Circ. Arrhythm. Electrophysiol..

[82] Epstein A.E., Abraham W.T., Bianco N.R., Kern K.B., Mirro M., Rao S.V., Rhee E.K., Solomon S.D., Szymkiewicz S.J. (2013). Wearable Cardioverter-Defibrillator Use in Patients Perceived to be at High Risk Early Post-Myocardial Infarction. J. Am. Coll. Cardiol..

[83] Cheung C.C., Olgin J.E., Lee B.K. (2021). Wearable cardioverter-defibrillators: A review of evidence and indications. Trends Cardiovasc. Med..

[84] Adler A., Halkin A., Viskin S. (2013). Wearable Cardioverter-Defibrillators. Circulation.

[85] Garcia R., Combes N., Defaye P., Narayanan K., Guedon-Moreau L., Boveda S., Blangy H., Bouet J., Briand F., Chevalier P. (2021). Wearable cardioverter-defibrillator in patients with a transient risk of sudden cardiac death: The WEARIT-France cohort study. Europace.

[86] Kondo Y., Linhart M., Andrié R.P., O Schwab J. (2015). Usefulness of the wearable cardioverter defibrillator in patients in the early post-myocardial infarction phase with high risk of sudden cardiac death: A single-center European experience. J. Arrhythm..

[87] Hioki H., Kozuma K., Kobayashi Y., Ando K., Morino Y., Kishihara J., Ako J., Ikari Y. (2022). Wearable cardioverter-defibrillators after myocardial infarction: A review of its clinical utility and unmet needs in current clinical practice. Cardiovasc. Interv. Ther..

[88] Kutyifa V., Moss A.J., Klein H., Biton Y., McNitt S., MacKecknie B., Zareba W., Goldenberg I. (2015). Use of the wearable cardioverter defibrillator in high-risk cardiac patients: Data from the Prospective Registry of Patients Using the Wearable Cardioverter Defibrillator (WEARIT-II Registry). Circulation.

[89] Olgin J.E., Pletcher M.J., Vittinghoff E., Wranicz J., Malik R., Morin D.P., Zweibel S., Buxton A.E., Elayi C.S., Chung E.H. (2018). Wearable cardioverter-defibrillator after myocardial infarction. N. Engl. J. Med..

[90] Miller J.D., Yousuf O., Berger R.D. (2015). The implantable cardioverter-defibrillator: An update. Trends Cardiovasc. Med..

[91] Maron D.J., Hochman J.S., Reynolds H.R., Bangalore S., O’Brien S.M., Boden W.E., Chaitman B.R., Senior R., López-Sendón J., Alexander K.P. (2020). Initial Invasive or Conservative Strategy for Stable Coronary Disease. ISCHEMIA Research Group. N. Engl. J. Med..

[92] Bershtein L., Sumin A., Zbyshevskaya E., Gumerova V., Tsurtsumia D., Kochanov I., Andreeva A., Piltakian V., Sayganov S. (2023). Stable Coronary Artery Disease: Who Finally Benefits from Coronary Revascularization in the Modern Era? The ISCHEMIA and Interim ISCHEMIA-EXTEND Analysis. Life.

[93] Thomsen P.E.B., Jons C., Raatikainen M.J.P., Joergensen R.M., Hartikainen J., Virtanen V., Boland J., Anttonen O., Gang U.J., Hoest N. (2010). Long-Term Recording of Cardiac Arrhythmias with an Implantable Cardiac Monitor in Patients with Reduced Ejection Fraction After Acute Myocardial Infarction—The Cardiac Arrhythmias and Risk Stratification after Acute Myocardial Infarction (CARISMA) Study. Circulation.

[94] Hindricks G., Taborsky M., Glikson M., Heinrich U., Schumacher B., Katz A., Brachmann J., Lewalter T., Goette A., Block B. (2014). Implant-based multiparameter telemonitoring of patients with heart failure (IN-TIME): A randomised controlled trial. Lancet.

